# Cingulate Alpha-2A Adrenoceptors Mediate the Effects of Clonidine on Spontaneous Pain Induced by Peripheral Nerve Injury

**DOI:** 10.3389/fnmol.2017.00289

**Published:** 2017-09-12

**Authors:** Yong-Jie Wang, Zhen-Xing Zuo, Cheng Wu, Li Liu, Zhi-Hui Feng, Xiang-Yao Li

**Affiliations:** ^1^Center for Mitochondrial Biology and Medicine, Frontier Institute of Science and Technology, Xi’an Jiaotong University Xi’an, China; ^2^The Key Laboratory of Biomedical Information Engineering of the Ministry of Education, School of Life Science and Technology, Xi’an Jiaotong University Xi’an, China; ^3^Department of Surgery, Tongji Hospital, School of Medicine, Tongji University Shanghai, China; ^4^Department of Physiology, Institute of Neuroscience, School of Medicine, Zhejiang University Hangzhou, China; ^5^Core Facility of School of Medicine, Zhejiang University Hangzhou, China

**Keywords:** spontaneous pain, peripheral nerve injury, α_2A_ adrenoceptors, clonidine, anterior cingulate cortex, stage-dependance, conditioned place preference, mechanical allodynia

## Abstract

The anterior cingulate cortex (ACC) is an important brain area for the regulation of neuropathic pain. The α_2A_ adrenoceptor is a good target for pain management. However, the role of cingulate α_2A_ adrenoceptors in the regulation of neuropathic pain has been less studied. In this study, we investigated the involvement of cingulate α_2A_ adrenoceptors in the regulation of neuropathic pain at different time points after peripheral nerve injury in mice. The application of clonidine, either systemically (0.5 mg/kg intraperitoneally) or specifically to the ACC, increased paw withdrawal thresholds (PWTs) and induced conditioned place preference (CPP) at day 7 after nerve injury, suggesting that cingulate α_2_ adrenoceptors are involved in the regulation of pain-like behaviors. Quantitative real-time PCR data showed that α_2A_ adrenoceptors are the dominant α_2_ adrenoceptors in the ACC. Furthermore, the expression of cingulate α_2A_ adrenoceptors was increased at day 3 and day 7 after nerve injury, but decreased at day 14, while no change was detected in the concentration of adrenaline or noradrenaline. BRL-44408 maleate, a selective antagonist of α_2A_ adrenoceptors, was microinfused into the ACC. This blocking of cingulate α_2A_ adrenoceptors activity abolished the CPP induced by clonidine (0.5 mg/kg intraperitoneally) but not the effects on PWTs at day 7. However, clonidine applied systemically or specifically to the ACC at day 14 increased the PWTs but failed to induce CPP; this negative effect was reversed by the overexpression of cingulate α_2A_ adrenoceptors. These results suggest that cingulate α_2A_ adrenoceptors are necessary for the analgesic effects of clonidine on spontaneous pain.

## Introduction

Neuropathic pain refers to the pain caused by injury or disease in the somatosensory nerve system; it affects 5%–8% of the population in the human society (Colloca et al., 2017). Most patients with neuropathic pain suffer from spontaneous pain (Backonja and Stacey, 2004), which is pain felt in the absence of stimulation, as well as allodynia and hyperalgesia (Bennett, 2012; Tappe-Theodor and Kuner, 2014). The α_2A_ adrenoceptor is an important target for chronic pain treatments (Pertovaara, 2006, 2013). Binding of adrenaline or noradrenaline to the α_2A_ adrenoceptor inhibits the activities of adenylyl cyclase, therefore decreasing the concentration of cyclic adenosine monophosphate (cAMP). In the spinal cord, the activities of α_2_ adrenoceptors decrease excitatory synaptic transmission via the regulation on glutamate release. Clonidine, an agonist of α_2_ adrenoceptors, showed significant analgesic effects on both spontaneous and evoked pain (King et al., 2009), and topical clonidine is an option for the treatment of neuropathic pain (Finnerup et al., 2015).

The involvement of the anterior cingulate cortex (ACC) in the regulation of chronic pain has been widely studied (Bliss et al., 2016). The ACC is activated by acute noxious stimulations (Tang et al., 2005), and enhanced excitatory synaptic transmissions have been reported after peripheral nerve injury (Xu et al., 2008) or peripheral inflammation (Zhao et al., 2006). Increased activity of a brain-specific isoform of protein kinase C, zeta type (PKMζ) in the ACC was implicated in the regulation of both spontaneous and evoked pain (Li et al., 2010; King et al., 2012). Lesion of the ACC eliminated the place preference induced lidocaine in the rostral ventromedial medulla (RVM; Qu et al., 2011), suggesting that the ACC has a special role in the regulation of spontaneous pain. However, whether the α_2A_ adrenoceptors in the ACC are involved in the regulation of neuropathic pain is largely unknown. Here, we investigated the role of cingulate α_2A_adrenoceptors in the regulation of both evoked pain and spontaneous pain induced by peripheral nerve injury. Our data, at both the behavioral and the molecular level, suggest that cingulate α_2A_ adrenoceptors are important for the regulation of spontaneous pain. We also found that the expression of cingulate α_2A_ adrenoceptors was different at day 7 and day 14 after common peroneal nerve (CPN) ligation, which led to different analgesic effects of clonidine on spontaneous pain; therefore, our data suggest that analgesic effects on may be differential according to the developmental stage of neuropathic pain among in the clinic.

## Materials and Methods

### Animals

C57BL/6 male mice aged 8–10 weeks (20–35 g) were used in this study; animals were housed 4 or 5 per cage at constant room temperature (25 ± 1°C) and relative humidity (60 ± 5%) under a 12 h light/dark schedule (light 07.00–19:00), and food and water were available* ad libitum*. For the behavioral tests, the mice were allowed to adapt to laboratory conditions for about 1 week and to habituate to the testing situation for at least 15 min before experiments. The animal care and use committee of Zhejiang University, China, approved all of the mouse protocols (No. ZJU2015-177-01). The material data safety sheets (MSDS) of chemical products, reagents and antibodies were followed during the experiments. Biohazard wastes were managed following the Medical Waste Management Regulations of China.

### CPN Model

The common peroneal nerve (CPN) ligation mouse model of neuropathic pain has been described previously (Vadakkan et al., 2005; Li et al., 2010). Briefly, mice were anesthetized using isoflurane (1%–3%, as needed). The left CPN between the anterior and posterior groups of muscles was ligated slowly with 5–0 chromic gut suture (Ethicon), until twitching of the digits was observed. The skin was sutured using 5–0 silk suture and cleaned with povidone iodine. Sham surgery was conducted in the same manner, but the nerve was not ligated. All animals were kept in a normal living chamber post-surgery.

### Mechanical Allodynia Test

On the experimental day, the von Frey behavioral assay was performed according to the up-down algorithm described by Chaplan et al. (1994). To determine evoked reflex responses to mechanical stimuli, animals were placed on a raised mesh grid and covered with a clear plastic box for containment. Calibrated von Frey filaments were applied to the middle of the plantar surface of each paw until the filament bent. Brisk withdrawal or paw flinching was considered to be a positive response. Lifting of the paw due to normal locomotor behavior was ignored. In the absence of a response, the filament of the next greater force was applied. Following a response, the filament of the next lower force was applied. The tactile stimulus producing a 50% likelihood of withdrawal response was calculated and taken as the paw withdrawal threshold (PWT).

### Conditioned Place Preference Test

Conditioned place preference (CPP) was adapted from the behavioral paradigm established by King et al. (2009, 2012) in adult rats. Briefly, mice were pre-conditioned for 3 days: the mice were allowed to explore the chamber freely for 30 min on the first 2 days, their behaviors were recorded by a video camera on the 3rd day, and the time spent in each chamber was recorded. The following day, mice received the appropriate control (i.e., vehicle) paired with a randomly chosen chamber in the morning, and the appropriate drug treatment paired with the other chamber 4 h later (in the afternoon). Twenty hours following the afternoon pairing, mice were placed in the CPP box with access to all chambers; their behavior was recorded for 15 min and the recordings were analyzed for chamber preference. The preference index (PI) was calculated as the time spent in the drug-paired chamber subtracted from the time spent in the saline-paired chamber.

### Cannulation and Microinjection

The cannula surgery and microinjection were performed as described previously (Li et al., 2010). Briefly, mice were anesthetized using isoflurane (1%–3%, as needed) in 100% oxygen at 0.5 L/min via a facemask. The scalp was shaved and cleaned with povidone iodine (Triadine, Shanghai, China) and alcohol. The head was fixed into an adapter mounted on a stereotaxic frame (model 962; Kopf, California, CA, USA) and AKWA Tears (Akorn, Buffalo Grove, IL, USA) was applied to the eyes. An incision was made over the skull and the surface was exposed. Two small holes were drilled above the ACC, and the dura was gently reflected. Guide cannulas were placed 0.7 mm anterior to the Bregma, 0.3 mm lateral to the midline and 0.75 mm ventral to the surface of the skull. For microinjection, each mouse was restrained in a plastic cone (Braintree Scientific, Braintree, MA,USA), and a small hole was cut in the plastic overlying the microinjection guides. Each dummy cannula was removed, and a microinjection cannula was inserted into each guide. A 30-gauge injection cannula was inserted to a depth of 0.7 mm deeper than each guide. Clonidine (0.5 μl, 8 μg/μl) or BRL-44408 maleate (0.5 μl, 10 μg/μl) was delivered at 0.5 μl/min using a syringe driven by an infusion pump (Harvard Apparatus, Inc., South Natick, MA, USA). After delivery to one side of the brain, the cannula was left in place for 1 min to prevent solution from flowing back up the guide. The cannula was then retracted and inserted into the opposite side of the brain. Ten minutes after microinjection, the mice were put into the chamber for the CPP conditioning or given the mechanical allodynia test.

### Real-Time PCR Assay

Reverse transcription (RT) was performed using a PrimeScript^®^ RT Reagent Kit with gDNA Eraser (TaKaRa, Dalian, China) in a 20 μl reaction mixture containing 1 μg of total RNA from each individual sample. The following primer pairs were used to detect the genes of interest (Table 1). qRT-PCR was carried out in a total volume of 25 μl, with each tube containing 12.5 μl of SYBR Premix Ex Taq (TaKaRa, Dalian, China), 4 μl of RT product (40 ng) and 2 μl of primers (400 nM each). Three replicates were conducted per sample. Reactions were run in Roche LightCycler 480II at 95°C for 3 min and then 40 cycles at 95°C for 5 s and 60°C for 15 s. The qRT-PCR experiments were repeated three times. We obtained the ratios between the genes of interest and β-actin to calculate the relative abundance of mRNA levels in each sample. Relative quantification of the mRNA was calculated by the comparative CT method (2^−ΔΔCT^ method).

**Table 1:** Primers for RT-qPCR.

Primer names	Sequences (5′-3′)
Adar2A-F	GCCATCATTGTCACCGTGTGGGT
Adar2A-R	CTGGTCGTTGATCTTGCAGCTTG
Adra2B-F	CGATGAGACCTGGTACATCTTGTCC
Adra2B-R	GCCTTGCCCAGCCCATTCT
Adra2C-F	CGTGCGTGGTGCGAGGTCTA
Adra2C-R	TTTGATGCGGCGTGGAGTGC
actin-F	AGACTTCGAGCAGGAGATGGC
actin-R	TCGTTGCCAATAGTGATGACCTG

*RT-qPCR, reverse transcriptase quantitative polymerase chain reaction; F, Forward; R, Reverse*.

### Western Blot Analysis

The mice were lightly anesthetized with isoflurane and then decapitated. The regions of ACC was dissected after 3 days, 7 days and 14 days of CPN ligation and then homogenized in an RIPA buffer (50 mM pH 7.6 Tris-Cl, 150 mM NaCl, 1 mM EDTA, 1% NP-40, 0.1% SDS, 1 mM DTT, 0.5% sodium deoxycholate) containing a protease inhibitor cocktail. After centrifugation, the supernatants were used for protein quantification by the Bradford assay. Electrophoresis of equal amounts of total protein was performed on SDS-polyacrylamide gels. The separated proteins were transferred onto polyvinylidene membranes at 4°C. The membranes were blocked for 2 h with 5% milk in TBST (Tris-buffered saline with Tween 20, room temperature) and incubated with a primary antibody (Adra2a 1:200, Proteintech) at 4°C overnight. After being washed, the membranes were incubated with the appropriate HRP-coupled secondary antibody (Beyotime, China) diluted 1:1000 for 1 h, followed by enhanced chemiluminescence detection of the proteins with Western lightning Chemiluminescence Reagent Plus, according to the manufacturer’s instructions. To verify equal loading, we also probed the membranes with antibodys against tubulin (1:10,000 Sigma) or actin (1:5000, Sigma). The density of the immunoblots was measured with the NIH ImageJ program.

### Constructs, Viral Packaging and Stereotactic Injection

The adeno-associated virus (AAV) Adra2a was constructed and packaged by Vigene Biosciences (Jinan, China). The coding sequence of mouse α_2A_ adrenoreceptor (Adra2a) was synthesized and cloned into pAAV9–cytomegalovirus (CMV)–mCherry plasmid to produce pAAV9–CMV–Adra2a–P2A–mCherry. AAV9–CMV–Adra2a–P2A–mCherry was produced by transfection of AAV-293 cells with pAAV9–CMV–Adra2a–P2A–mCherry, AAV helper plasmid (pAAV Helper), and AAV Rep/Cap expression plasmid. Viral particles were purified by an iodixanol step-gradient ultracentrifugation method. The genomic titer was 1.1 × 10^12^ genomic copies per ml determined by quantitative PCR.

For viral injection, mice were anesthetized with ketamine (100 mg/kg of body weight) and xylazine (8 mg/kg) by intraperitoneal (IP) injection and placed in a stereotactic frame. Mice were injected bilaterally with purified and concentrated AAV into the ACC (1 μl, coordinates from Bregma: −0.7 mm anterior/posterior, ±0.3 mm medial/lateral, −1.75 mm dorsal/ventral) using glass microelectrodes at a slow rate (100 nl/min). The injection microelectrode was slowly withdrawn 10 min after the virus infusion. The CPN ligation was carried out on day 8 after virus injection, and the CPP experiment was started from day 10 after the ligation, to ensure the test could be performed at day 14. The injection sites were examined at the end of all the behavioral tests, and only data from animals with correct injections were included. Brain slices of ACC were directly examined under a fluorescent microscope. The PWTs were examined in a blind manner.

### Data Analysis

SigmaPlot version 11.0 was used to plot and fit the data. Statistical comparisons were made using the student’s *t*-test, paired *t*-test, and one-or two-way repeated measures (RM) analysis of variance (ANOVA). The Student–Newman–Keuls test was used for *post hoc* comparison. All data are presented as the mean ± standard error of the mean (SEM). In all cases, *P* < 0.05 was considered statistically significant.

## Results

### Spontaneous Pain Induced by Common Peroneal Nerve Ligation

**Figure 1:**
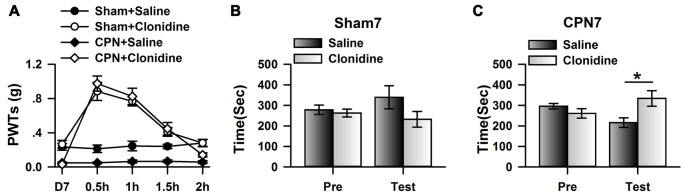
Clonidine induced place preference at day 7 after common peroneal nerve (CPN). **(A)** The application of clonidine (i.p. 0.5 mg/kg) increased the paw withdrawal threshold (PWT) of mice with sham or CPN treatments at day 7. For the saline injection experiments: two-Way repeated measures (RM) analysis of variance (ANOVA), Sham vs. Injury: *F*_(1,79)_ = 48.74, *P* < 0.01, Time: *F*_(4,79)_ = 0.93, *P* > 0.05, interaction: *F*_(4,79)_ = 0.58, *P* > 0.05, *n* = 6 for sham, *n* = 10 for CPN group. For the clonidine injection experiments: two-Way RM ANOVA, Sham vs. Injury: *F*_(1,69)_ = 0.74, *P* > 0.05, Time: *F*_(4,69)_ = 46.45, *P* < 0.01, interaction: *F*_(4,69)_ = 1.92, *P* > 0.05, *n* = 6 for sham, *n* = 8 for CPN group. **(B)** The mice from the sham group did not show the place preference at day 7 after CPN. two-Way RM ANOVA, Pre vs. Test: *F*_(1,23)_ = 0.72, *P* > 0.05, saline vs. clonidine: *F*_(1,23)_ = 1.01, *P* > 0.05, interaction: *F*_(1,23)_ = 1.97, *P* > 0.05, *n* = 6 per group. **(C)** The mice with CPN ligation spent longer time in the clonidine paired chamber than the saline paired chambers at day 7 after CPN. Two-Way RM ANOVA, Pre vs. Test: *F*_(1,27)_ = 0.04, *P* > 0.05, saline vs. clonidine: *F*_(1,27)_ = 0.97, *P* > 0.05, interaction: *F*_(1,27)_ = 29.97, *P* < 0.05, *n* = 7, *P < 0.05.

Previously, it has been shown that CPN ligation induced maladaptive plasticity in the ACC, which may further mediate mechanical allodynia and spontaneous pain. Here, we examined the analgesic effects of clonidine (0.5 mg/kg, IP) on the mechanical allodynia and spontaneous pain induced by CPN ligation. As shown in Figure 1A, CPN ligation decreased the PWTs of left hindpaw, and the application of clonidine increased the PWTs of mice with sham treatments or CPN ligation (Figure 1A), suggesting that the hypersensitivity was reversed by clonidine. The CPP behavioral paradigm has been widely used to examine the effects of various chemicals on the spontaneous pain. We further examined the effects of clonidine on place preference. As shown in Figure 1B, clonidine did not induce place preference in the sham group (pre vs. test: *F*_(1,23)_ = 0.72, *P* > 0.05; saline vs. clonidine: *F*_(1,23)_ = 1.01, *P* > 0.05; interaction: *F*_(1,23)_ = 1.97, *P* > 0.05; *n* = 6; two-way RM ANOVA), and the PI was similar between the pre-conditioning and testing periods (pre: −15.83 ± 40.61 s test: −107.17 ± 89.36 s *n* = 6; paired *t-test*, *P* > 0.05). However, clonidine did induce place preference in the mice with CPN ligation (pre vs. test: *F*_(1,27)_ = 0.04, *P* > 0.05; saline vs. clonidine: *F*_(1,27)_ = 0.97, *P* > 0.05; interaction: *F*_(1,27)_ = 29.97, *P* < 0.05; *n* = 7; two-way RM ANOVA; Figure 1C), *post hoc* comparison showed a significant difference between the time spent in the saline chamber and the clonidine chamber during the test period, and a significant difference was detected between the PI of the pre-conditioning period and that of the testing period (pre: −34.71 ± 33.75 s test: 117.57 ± 52.97 s paired *t*-test, *P* < 0.01). Therefore, the application of clonidine systemically alleviated both mechanical allodynia and spontaneous pain at day 7 after CPN ligation.

### Microinjection of Clonidine into the ACC Induced CPP at Day 7

**Figure 2:**
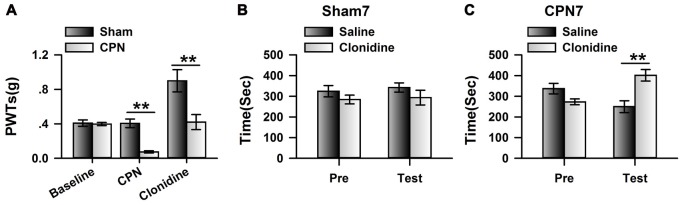
Microinfusion of clonidine into anterior cingulate cortex (ACC) induced place preference at day 7 after CPN. **(A)** Microinfusion of Clonidine into ACC increased the PWTs of mice from sham group or CPN group. Two-Way RM ANOVA, sham vs. injury: *F*_(1,47)_ = 21.05, *P* < 0.05, treatments: *F*_(2,47)_ = 22.97, *P* < 0.05, interaction: *F*_(2,47)_ = 7.37, *P* < 0.05, *n* = 7 for sham group, *n* = 9 for CPN group. **P < 0.01. **(B)** Microinfusion of clonidine into ACC of mice with sham treatments failed to induce the conditioned place preference (CPP) at day 7 after sham operation. Two-Way RM ANOVA, Pre vs. Test: *F*_(1,27)_ = 0.08, *P* > 0.05, saline vs. clonidine: *F*_(1,27)_ = 5.26, *P* > 0.05, interaction: *F*_(1,27)_ = 0.05, *P* > 0.05, *n* = 7. **(C)** Microinfusion of clonidine into ACC induced significant place preference on the mice with CPN ligation at day 7 after CPN. Two-Way RM ANOVA, Pre vs. Test: *F*_(1,23)_ = 3.32, *P* > 0.05, saline vs. clonidine: *F*_(1,23)_ = 2.39, *P* > 0.05, interaction: *F*_(1,23)_ = 22.60, *P* < 0.05, *n* = 6, **P < 0.01.

To further investigate the involvement of cingulate α_2_ receptors in pain regulation, we directly activated cingulate α_2_ adrenoceptors by microinjecting clonidine into the ACC. Similar to our previous observations (Zuo et al., 2015), clonidine increased the PWTs of both sham and CPN-ligated animals at day 7 (Figure 2A), suggesting that activating cingulate α_2_ adrenoceptors is sufficient to alleviate mechanical allodynia. Furthermore, the sham-treated mice spent equal time in both chambers (pre vs. test: *F*_(1,27)_ = 0.08, *P* > 0.05; saline vs. clonidine: *F*_(1,27)_ = 5.26, *P* > 0.05; interaction: *F*_(1,27)_ = 0.05, *P* > 0.05; *n* = 7; two-way RM ANOVA; Figure 2B), and there was no difference between the PIs of the pre-conditioning and test periods (pre: −48.83 ± 24.69 s test: −25.28 ± 23.21 s paired *t*-test, *P* > 0.05). However, the mice with CPN ligation spent a longer time in the clonidine-paired chamber (pre vs. test: *F*_(1,23)_ = 3.32, *P* > 0.05; saline vs. clonidine: *F*_(1,23)_ = 2.39, *P* > 0.05; interaction: *F*_(1,23)_ = 22.60, *P* < 0.05; *n* = 6; two-way RM ANOVA; Figure 2C); *post hoc* comparison showed a significant difference between the time spent in the saline chamber and the clonidine chamber during the test period (*P* < 0.01); and, consistently, the PI was significantly different at day 7 (pre: −64.07 ± 28.87 s test: 151.75 ± 36.41 s paired *t*-test, *n* = 6, *P* < 0.01). These data indicate that activating the cingulate α_2_ adrenoceptors is sufficient to alleviate both evoked pain and spontaneous pain at day 7 after nerve injury.

### The Expression of α_2A_ Adrenoceptors in the ACC after Nerve Injury

**Figure 3:**
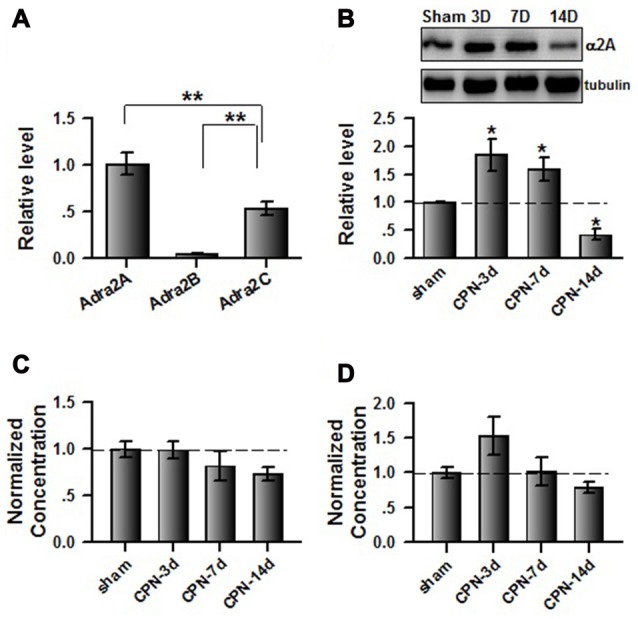
Peripheral nerve injury changed the expression of cingulate α_2A_ adrenoceptors. **(A)** Summarized data to show the relative mRNA level of α_2A_, α_2B_ and α_2C_ adrenoceptors in the ACC. One-Way ANOVA, *F*_(2,8)_ = 35.41, *P* < 0.001, *n* = 3 for each group. **P < 0.01. **(B)** The CPN ligation increased the expression of cingulate α_2A_ adrenoceptors at day 3 and day 7, but decreased at day 14. One-Way ANOVA, *F*_(3,19)_ = 12.58, *P* < 0.001, *n* = 5 for each group. *P < 0.05. **(C)** The CPN ligation did not change the concentration of adrenaline in the ACC, One-way ANOVA, *F*_(3,19)_ = 1.53, *P* > 0.05, *n* = 5 per group. **(D)** The CPN ligation did not change the concentration of noradrenaline in the ACC, One-way ANOVA, *F*_(3,19)_ = 3.19, *P* > 0.05, *n* = 5 per group.

As different subtypes of α_2_ adrenoceptors are expressed in the central nervous system, we used quantitative real-time PCR to examine the mRNA levels of *Adra2A*(*α_2A_*), *Adra2B*(*α_2B_*) and *Adra2C*(*α_2C_*) in the ACC. Interestingly, we found that the relative level of mRNA for *Adra2A* (1.01 ± 0.12) is significantly higher than for *Adra2C* (0.53 ± 0.07) or* Adra2B* (0.04 ± 0.01; one-way ANOVA, *F*_(2,8)_ = 35.41, *P* < 0.001, *n* = 3 per group; Figure 3A). Previously, Mika Scheinin’s group reported that clonidine has significant lower Ki value to the α_2A_ (17.2 ± 1.54) than α_2B_ (56.0 ± 12.6) and α_2C_ (96.9 ± 8.7), suggesting that the affinity of clonidine to *α*_2A_ adrenoceptor is higher than others (Pohjanoksa et al., 1997), we decided to focus on the involvement of cingulate α_2A_ adrenoceptors in pain regulation. We further examined the expression of α_2A_ adrenoceptors in the ACC at different time points after peripheral nerve injury. Notably, we observed an increased expression of α_2A_ receptors at day 3 (1.85 ± 0.29 times; *n* = 5) and day 7 (1.59 ± 0.22 times; *n* = 5) compared with sham treatments (Figure 3B). Surprisingly, α_2A_ receptor expression was downregulated at day 14 (0.42 ± 0.10 times; *n* = 5) after nerve injury. In addition, one-way ANOVA showed that time has significant effects on the expression of α_2A_ adrenoceptors (*F*_(3,19)_ = 12.58, *P* < 0.001). As the activities of α_2A_ receptors depend on the binding of their endogenous ligands, we further examined the concentrations of adrenaline and noradrenaline in the ACC. Unlike the expression of α_2A_ receptors, the concentrations of adrenaline (one-way ANOVA, *F*_(3,19)_ = 1.53, *P* > 0.05; Figure 3C) and noradrenaline (one-way ANOVA, *F*_(3,19)_ = 3.19, *P* > 0.05; Figure 3D) were not changed at the tested time points after nerve injury. Therefore, the increased α_2A_ receptors at day 3 and day 7 may not be fully activated owing to insufficient endogenous ligands.

### Blocking the Activities of α_2A_ Adrenoceptors Abolished the Effects of Clonidine on Spontaneous Pain on Day 7

**Figure 4:**
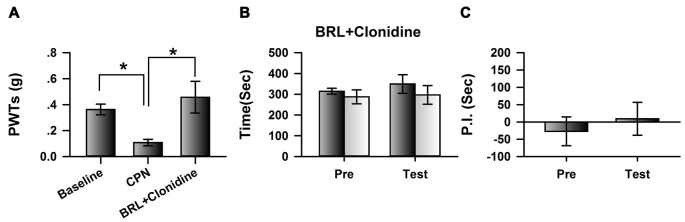
Micro-infusion of BRL-44408 maleate into ACC blocked the effects of clonidine on the CPP at day 7 after CPN. **(A)** BRL-44408 maleate in the ACC did not block the effects of clonidine (i.p. 0.5 mg/kg) on the PWTs. One-Way RM ANOVA, *F*_(2,20)_ = 6.16, *P* < 0.05, *n* = 7. *P < 0.05. **(B)** Clonidine did not induce the place preference when the BRL-44408 maleate was infused in the ACC of mice with sham treatments. Two-Way RM ANOVA, Pre vs. Test: *F*_(1,23)_ = 5.68, *P* = 0.06, saline vs. clonidine: *F*_(1,23)_ = 0.59, *P* > 0.05, interaction: *F*_(1,23)_ = 0.08, *P* > 0.05, *n* = 6. **(C)** Clonidine did not induce the place preference at day 7 when the BRL-44408 maleate was infused in the ACC of mice with nerve injury. Paired *t*–test, *P* > 0.05.

We further blocked the activities of cingulate α_2A_ adrenoceptors by microinfusion of BRL-44408 maleate, a selective α_2A_ adrenoceptor antagonist, into the ACC at day 7 after nerve injury, and further examined the analgesic effects of clonidine (0.5 mg/kg IP). Clonidine still increased the PWTs of CPN mice after the BRL-44408 maleate infusion (Figure 4A). However, when BRL-44408 maleate was applied to the ACC, clonidine failed to induce place preference on the mice with nerve injury (pre vs. test: *F*_(1,23)_ = 5.68, *P* = 0.06; saline vs. clonidine: *F*_(1,23)_ = 0.59, *P* > 0.05; interaction: *F*_(1,23)_ = 0.08, *P* > 0.05; *n* = 6; two-way RM ANOVA; Figure 4B). No difference was detected between the PI of the pre-conditioning period and that of the testing period (Figure 4C). Therefore, the cingulate α_2A_ adrenoceptors are necessary for the analgesic effects of clonidine on spontaneous pain.

### Microinjection of Clonidine into the ACC Failed to Induce CPP at Day 14

**Figure 5:**
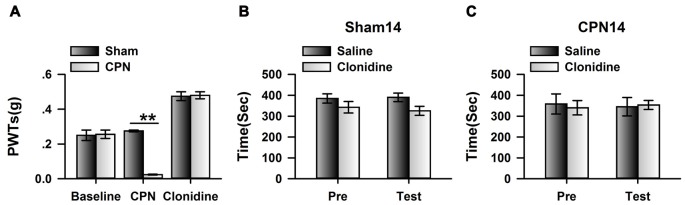
Micro-infusion of clonidine into ACC failed to induce place preference at day 14 after CPN. **(A)** Micro-infusion of clonidine into ACC of mice increased the PWTs of mice from both groups at day 14. Two-Way RM ANOVA, Sham vs. Injury: *F*_(1,26)_ = 19.23, *P* < 0.01, treatments: *F*_(2,26)_ = 154.09, *P* < 0.01, interaction: *F*_(2,26)_ = 30.05, *P* < 0.01, *n* = 4 for sham, *n* = 5 for CPN group. **P < 0.01. **(B)** The application of clonidine into the ACC failed to induce place preference on the sham mice at day 14. Two-Way RM ANOVA, Pre vs. Test: *F*_(1,15)_ = 0.29, *P* > 0.05, saline vs. clonidine: *F*_(1,15)_ = 2.37, *P* > 0.05, interaction: *F*_(1,15)_ = 0.34, *P* > 0.05, *n* = 4. **(C)** The application of clonidine to the ACC failed to induce place preference on the mice with CPN treatments at day14. Two-Way RM ANOVA, Pre vs. Test: *F*_(1,19)_ = 0.01, *P* > 0.05, saline vs. clonidine: *F*_(1,19)_ = 0.01, *P* > 0.05, interaction: *F*_(1,19)_ = 0.16, *P* > 0.05, *n* = 5.

If cingulate α_2A_ adrenoceptors are necessary for the regulation of spontaneous pain, we reasoned that clonidine may have a less effect on spontaneous pain at day 14 owing to downregulation of the α_2A_ adrenoceptors. We therefore microinjected the clonidine into the ACC at day 14 after nerve injury; similar to the observations at day 7, the application of clonidine increased the PWTs of mice with sham or CPN treatments (Figure 5A), suggesting the alleviation of mechanical allodynia induced by nerve injury. Similarly, clonidine failed to induce CPP in the sham-treated mice (Figure 5B). In line with our idea, activating the cingulate α_2A_ adrenoceptors failed to induce place preference at day 14 in the mice with CPN ligation (Figure 5C).

### Clonidine Applied Systemically did Not Induce Place Preference at Day 14 after Nerve Injury

**Figure 6:**
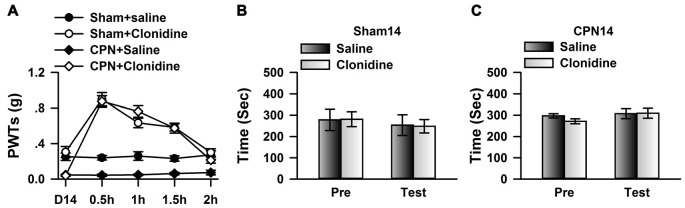
Clonidine failed to induce place preference at day 14 after CPN. **(A)** The application of clonidine (i.p. 0.5 mg/kg) increased the PWTs of mice with sham or CPN treatments at day 14. For the saline injection experiments: Two-Way RM ANOVA, Sham vs. Injury: *F*_(1,79)_ = 83.61, *P* < 0.01, Time: *F*_(4,79)_ = 0.52, *P* > 0.05, interaction: *F*_(4,79)_ = 0.23, *P* > 0.05, *n* = 6 for sham, *n* = 10 for CPN group. For the clonidine injection experiments: Two-Way RM ANOVA, Sham vs. Injury: *F*_(1,69)_ = 0.94, *P* > 0.05, Time: *F*_(4,69)_ = 77.60, *P* < 0.01, interaction: *F*_(4,69)_ = 4.47, *P* < 0.01, *n* = 6 for sham, *n* = 8 for CPN group. **(B)** Clonidine (0.5 mg/kg) failed to induce place preference on the mice with sham treatments at day 14 after sham operation. Two-Way RM ANOVA, Pre vs. Test: *F*_(1,19)_ = 1.82, *P* > 0.05, saline vs. clonidine: *F*_(1,19)_ = 0.01, *P* > 0.05, interaction: *F*_(1,19)_ = 0.05, *P* > 0.05, *n* = 5. **(C)** Clonidine (0.5 mg/kg) failed to induce place preference on the mice with CPN ligation at day 14 after CPN. Two-Way RM ANOVA, Pre vs. Test: *F*_(1,39)_ = 7.48, *P* < 0.05, saline vs. clonidine: *F*_(1,39)_ = 0.62, *P* > 0.05, interaction: *F*_(1,39)_ = 1.57, *P* > 0.05, *n* = 10.

To further confirm the observations at day 14, we examined the effects of systemically applied clonidine on CPP at day 14 after CPN. Positive effects were observed on the PWTs of both the sham group and the CPN group (Figure 6A). Clonidine did not induce place preference in mice from the sham group observed by the spent time (pre vs. test: *F*_(1,19)_ = 1.82, *P* > 0.05; saline vs. clonidine: *F*_(1,19)_ = 0.01, *P* > 0.05; interaction: *F*_(1,19)_ = 0.05, *P* > 0.05; *n* = 5; two-way RM ANOVA; Figure 6B). Consistent with the previous observations, no place preference (pre vs. test: *F*_(1,39)_ = 7.48, *P* < 0.05; saline vs. clonidine: *F*_(1,39)_ = 0.62, *P* > 0.05; interaction: *F*_(1,39)_ = 1.57, *P* > 0.05; *n* = 10; two-way RM ANOVA; Figure 6C) was induced by clonidine (0.5 mg/kg IP) in the CPN-ligated group at day 14.

### Overexpressing Cingulate α_2A_ Adrenoceptors Changed CPP Performance at Day 14

**Figure 7:**
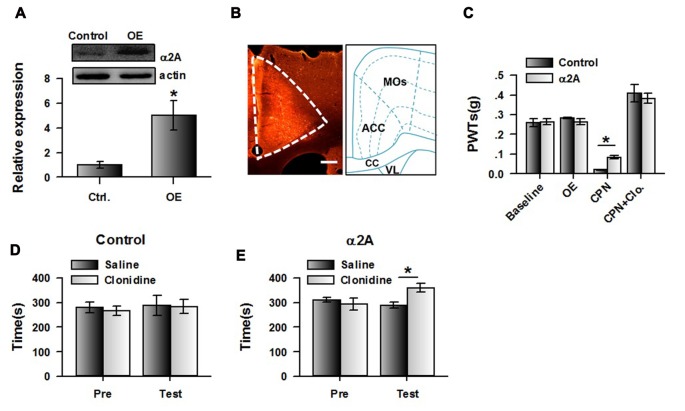
Over-expressing of α_2A_ adrenoceptors alleviated the evoked pain tested at day 14 after CPN. **(A)** The representative immunoblotting bands (top) and the quantitative data showed that the expression of α_2A_ adrenoceptors was increased in ACC with AAV9-CMV-Adra2a-P2A-mCherry, but not its control. *t*-test, *P* < 0.05, *n* = 4 per group, *P < 0.05. **(B)** An example of the virus expression in ACC. **(C)** The PWTs were slightly increased by the overexpressed α_2A_ adrenoceptors in the ACC at day 14 after nerve injury. Two-Way RM ANOVA, control vs. virus: *F*_(1,55)_ = 0.16, *P* > 0.05, treatments: *F*_(3,55)_ = 98.15, *P* < 0.05, interaction: *F*_(3,55)_ = 1.83, *P* > 0.05, *n* = 6 for control group, *n* = 8 for α_2A_ virus injection group. *P < 0.05. **(D)** The mice with the control virus injection did not show the place preference at day 14 after CPN. Two-Way RM ANOVA, Pre vs. Test: *F*_(1,23)_ = 0.44, *P* > 0.05, saline vs. clonidine: *F*_(1,23)_ = 0.04, *P* > 0.05, interaction: *F*_(1,23)_ = 0.07, *P* > 0.05, *n* = 6. **(E)** Clonidine (0.5 mg/kg) induced place preference at day 14 after CPN on the mice with over-expressed of α_2A_ adrenoceptors in the ACC. *P < 0.05. Two-Way RM ANOVA, Pre vs. Test: *F*_(1,31)_ = 4.10, *P* > 0.05, saline vs. clonidine: *F*_(1,31)_ = 1.69, *P* > 0.05, interaction: *F*_(1,31)_ = 14.92, *P* < 0.05, *n* = 8.

If our hypothesis is correct, increasing expression of α_2A_ adrenoceptor in the ACC may change the effects of clonidine on CPP at day 14. We therefore expressed the AAV with α_2A_ adrenoceptor overexpression into the ACC. Eight days after the virus injection, CPN ligation was performed. Mechanical allodynia and CPP were tested 14 days after nerve ligation. We indeed found that at day 14 after CPN, the expression of α_2A_ adrenoceptors in the ACC was increased in the virus-injected group compared with the control group, using western blot (Figure 7A) and immunofluorescence approaches (Figure 7B). The PWTs of virus-injected mice were also decreased by the nerve injury, although they were still slightly higher than those of the control group (control: 0.02 ± 0.01 g, *n* = 6; α_2A_: 0.08 ± 0.01 g, *n* = 8; *t*-test, *P* < 0.01; Figure 7C), and the application of clonidine (0.5 mg/kg) significantly increased the PWTs of both the control group and the group with overexpression of α_2A_ adrenoceptors. These results indicate that activating α_2A_ adrenoceptors may alleviate mechanical allodynia.

Interestingly, clonidine did not induce place preference in the control group (pre vs. test: *F*_(1,23)_ = 0.44, *P* > 0.05; saline vs. clonidine: *F*_(1,23)_ = 0.04, *P* > 0.05; interaction: *F*_(1,23)_ = 0.07; *n* = 6; two-way RM ANOVA; Figure 7D), and the PI was similar between groups (pre: −13.65 ± 32.29 s test: −3.53 ± 55.88 s paired *t*-test, *P* > 0.05). Conversely, we found that clonidine was able to induce CPP in the CPN-ligated mice with overexpression of α_2A_ adrenoceptors at day 14. As shown in Figure 7E, a longer time was spent in the clonidine-paired chamber during the testing period than the pre-conditioning period (pre vs. test: *F*_(1,31)_ = 4.10, *P* > 0.05; saline vs. clonidine: *F*_(1,31)_ = 1.69, *P* > 0.05; interaction: *F*_(1,31)_ = 14.92, *P* < 0.05; *n* = 6; two-way RM ANOVA; Figure 7E), and *post hoc* comparison showed a significant difference in the time spent in the saline chamber and the clonidine chamber during the test period. Consistently, a significant difference was detected in the PIs of the pre-conditioning and testing period (pre: −16.87 ± 25.19 s test: 71.37 ± 22.54 s paired *t-test*, *P* < 0.01). Therefore, the increased expression of cingulate α_2A_ adrenoceptors reversed the negative effects of clonidine on spontaneous pain at day 14.

## Discussion

In the current study, the involvement of cingulate α_2A_ adrenoceptors in the regulation of neuropathic pain was investigated using multiple approaches. We found that the expression of cingulate α_2A_ adrenoceptors was increased at day 3 and day 7 but decreased at day 14 after nerve injury; locally activating cingulate α_2A_ adrenoceptors at day 7 increased PWTs and induced significant place preference, whereas blocking cingulate α_2A_ adrenoceptors abolished the effects of clonidine (IP) on spontaneous pain but not mechanical allodynia. At day 14, activating cingulate α_2A_ adrenoceptors increased PWTs but failed to induce place preference; consistently, systemically applied clonidine alleviated mechanical allodynia but had no effects on spontaneous pain, which was rescued by the overexpression of cingulate α_2A_ adrenoceptors. Overall, our data strongly suggest that cingulate α_2A_ adrenoceptors are necessary for the analgesic effects of clonidine on spontaneous pain. Furthermore, from a developmental viewpoint, our results may explain the different analgesic effects of clonidine on different patients.

### The Role of Cingulate α_2A_ Adrenoceptors in the Regulation of Spontaneous Pain

The α_2A_ adrenoceptors are important targets for pain treatments. Clinically, intrathecal or epidural clonidine alone relieves chronic pain with a single shot (Glynn et al., 1988; Carroll et al., 1993; Siddall et al., 1994; Glynn and O’Sullivan, 1996), whereas in another trial, long-term infusion failed in about 81.82% (9/11) of participants (Ackerman et al., 2003). Recently, it was reported that intrathecal clonidine led to a more than 30% reduction in pain (Rauck et al., 2015). In preclinical studies, the analgesic effects of clonidine on spontaneous pain have been observed in rats with spinal nerve ligation (SNL; King et al., 2009) and spinal cord injury (Davoody et al., 2011), and in mice with chronic inflammatory pain, SNL (He et al., 2012), or CPN ligation (Zuo et al., 2015). The current study focused on both the necessity and the sufficiency of cingulate α_2A_ adrenoceptors for the regulation of neuropathic pain.

The ACC mediates the negative affective component of chronic pain (Shackman et al., 2011). Using positron emission tomography, covariation between the intensity of spontaneous pain and regional cerebral blood flow has been observed in the ACC and insular cortex in patients with mononeuropathy (Petrovic et al., 1999). Similarly, increased neuronal activities in the medial prefrontal cortex (including the rostral anterior cingulate) were detected during a sustained high-pain period in patients with chronic back pain (Baliki et al., 2006). Other studies applied various chemicals, including ζ-pseudosubstrate inhibitory peptide (ZIP; Li et al., 2010), IEM1460 (Liu et al., 2015) and clonidine (Zuo et al., 2015), to the ACC locally, and unmasking effects on the aversion induced by peripheral nerve injury were observed, whereas lesion (Qu et al., 2011) or injection of brain-derived neurotrophic factor (BDNF)–tropomyosin receptor kinase B (TrkB) antagonist into the rostral ACC completely blocked the CPP induced by clonidine (Zhang et al., 2016), suggesting that the ACC is necessary for the regulation of spontaneous pain. In the current study, we showed that application of clonidine topically to the ACC is sufficient to alleviate both mechanical allodynia and spontaneous pain at day 7 after nerve injury, and that inhibiting the activities of cingulate α_2A_ adrenoceptors abolishes the place preference induced by clonidine in CPN-ligated mice, suggesting that the activation of cingulate α_2A_ adrenoceptors is necessary for the analgesic effects of clonidine on the spontaneous pain.

### The Analgesic Effects of Clonidine at Different Time Points

The involvement of cingulate α_2A_ adrenoceptors in the regulation of spontaneous pain changed at different stages. In the early stages, such as day 3 and day 7 after nerve injury, application of clonidine—either locally to the ACC or systemically—unmasked the aversion effects induced by nerve injury, an effect that was not observed at day 14. These results were due to the change in expression levels of cingulate α_2A_ adrenoceptors, which were increased at day 7, but decreased at day 14. It has also been found that enhanced excitatory synaptic transmission in the ACC is involved in the maintenance of neuropathic pain (Zhao et al., 2006; Xu et al., 2008), and a higher level of phosphorylated PKMζ is detectable on days 3 and 7, while the protein level increases only on day 3 but not day 7 after nerve ligation (Li et al., 2010). By using the long-term two-photo imaging approach, the Nabekura group found remodeling of synapses in the primary somatosensory cortex induced by peripheral nerve injury at the early phase, which was associated with the development of neuropathic pain (Kim and Nabekura, 2011), and spine motility in the primary somatosensory cortex differed between the developmental and maintenance phases (Kim et al., 2011). The clinical results showed that task-related activities in the primary motor and associated cortex differed in the subacute and recovery periods after spinal cord injury (Jurkiewicz et al., 2007). Therefore, the neuronal mechanisms mediating neuropathic pain at ACC may be different at different stages, and temporal factors should be considered when clonidine is used to treat neuropathic pain in clinical settings.

### The Neuronal Mechanisms Mediating Evoked Pain and Spontaneous Pain

The neuronal mechanisms mediating evoked pain may be different from those involved in spontaneous pain (Tappe-Theodor and Kuner, 2014). The experience of pain includes both sensory and affective components (Li, 2013). There are some compounds that have analgesic effects in both spontaneous and evoked pain. For example, clonidine and ω-conotoxin have been found to alleviate both evoked pain and spontaneous pain (King et al., 2009), and ketamine significantly reduced ongoing pain and evoked pain (Gottrup et al., 2006). Interestingly, some compounds only showed effects on evoked pain, such as adenosine (King et al., 2009) on the allodynia induced by SNL, and huperzine A on the mechanical allodynia induced by CPN ligation (Zuo et al., 2015). These data strongly suggest that neuronal mechanisms mediating spontaneous pain and evoked pain are different (Hung et al., 2015). From a temporal viewpoint, our data provide new evidence regarding the differences between evoked pain and spontaneous pain. We showed that clonidine had analgesic effects on both mechanical allodynia and spontaneous pain at day 7 after CPN ligation, whereas it affects only mechanical allodynia at day 14. Furthermore, we found that inhibiting the activities of cingulate α_2A_ adrenoceptors abolished the effects of systemic clonidine on CPP, while it does not block the effects on mechanical allodynia. These data strongly suggest that cingulate α_2A_ adrenoceptors are mainly involved in the regulation of spontaneous pain, which could be alleviated by clonidine. However, further study is required to fully understand how cingulate α_2A_ adrenoceptors regulate spontaneous pain and evoked pain.

## Author Contributions

Y-JW and Z-XZ performed the CPP experiments and mechanical allodynia testing and performed the data analysis, and drafting the work. CW and LL performed the virus injection, analyzed the data. Z-HF and X-YL designed the experiments, analyzed the data and approved the draft, and X-YL wrote the article.

## Conflict of Interest Statement

The authors declare that the research was conducted in the absence of any commercial or financial relationships that could be construed as a potential conflict of interest.
